# Pre-Treatment Nutritional Status as a Predictor of Clinical Outcomes in Moderate-to-Severe Plaque Psoriasis Patients Undergoing Cyclosporine A Therapy

**DOI:** 10.3390/nu17193098

**Published:** 2025-09-29

**Authors:** Wojciech Kulej, Beniamin Oskar Grabarek, Martyna Stefaniak, Laura Opalska, Piotr Michalski, Aleksandra Plata-Babula, Anna Michalska-Bańkowska

**Affiliations:** 1 Collegium Medicum, WSB University, 41-300 Dabrowa Gornicza, Poland; martynastefaniakk@gmail.com (M.S.); laura.opalska@interia.pl (L.O.); piotrm703@gmail.com (P.M.); draleksandraplatababula@gmail.com (A.P.-B.); amgp1@o2.pl (A.M.-B.); 2 Faculty of Medicine and Health Sciences, Andrzej Frycz Modrzewski University in Kraków, 30-705 Kraków, Poland; 3 Faculty of Medicine, Academy of Silesia, 40-555 Katowice, Poland; 4 Individual Specialist Medical Practice Anna Michalska-Bańkowska, 41-253 Czeladź, Poland; 5 Department of Dermatology and Vascular Anomalies Treatment for Children, Faculty of Medicine in Katowice, Medical University of Silesia, 40-555 Katowice, Poland

**Keywords:** psoriasis, cyclosporine A, nutrition, dietary intake, nutrient deficiency, PASI, BSA, treatment outcomes

## Abstract

**Background/Objectives**: Psoriasis is a chronic immune-mediated disease frequently accompanied by systemic inflammation and metabolic disturbances. Nutrition plays a crucial role in modulating inflammatory pathways, yet the impact of baseline dietary status on systemic therapy outcomes remains underexplored. **Methods**: A total of 37 patients (20 men, 17 women; mean age 47.8 ± 4.87 years) scheduled for cyclosporine A (CsA) therapy underwent dietary assessment using 24 h recall and food frequency questionnaires. Intake was compared with dietary reference values. Psoriasis severity was measured by using the Psoriasis Area and Severity Index (PASI) and Body Surface Area (BSA) at baseline, day 42, and day 84. Mixed-effects regression models adjusted for body mass index (BMI), age, and sex assessed associations between nutrient adequacy and clinical outcomes. **Results**: Participants exhibited frequent dietary imbalances, including low polyunsaturated fatty acids, fiber, vitamin D, folate, and minerals such as magnesium and zinc, alongside excess saturated fat and sodium. Adequate intake of fiber, eicosapentaenoic acid (EPA)+ docosahexaenoic acid (DHA), and vitamins A and D, folate, magnesium, and zinc was independently associated with a lower baseline PASI/BSA and faster improvement during CsA therapy (*p* < 0.05). Higher BMI, older age, and male sex predicted poorer outcomes. **Conclusions**: Pre-treatment nutritional inadequacies are common in psoriasis and independently predict diminished therapeutic response to CsA. Early nutritional optimization may enhance treatment efficacy and support long-term disease control. Integrating dietary assessment in psoriasis management represents a feasible, impactful adjunct to pharmacotherapy.

## 1. Introduction

Psoriasis is a chronic, immune-mediated inflammatory disease that affects the skin and often extends its impact beyond dermatological symptoms, contributing to a systemic pro-inflammatory state [1]. The most common clinical form—plaque psoriasis—has been linked to an elevated risk of metabolic disorders, cardiovascular diseases, and reduced quality of life. Increasing evidence indicates that diet plays a significant role in modulating systemic inflammation, influencing both the course of psoriasis and patient response to treatment [2,3,4,5]. Nutritional factors such as excessive caloric intake, high saturated fat consumption, low intake of omega-3 polyunsaturated fatty acids, and inadequate levels of antioxidants may exacerbate inflammatory pathways, while healthier dietary patterns could have a protective effect [6,7,8,9,10].

Cyclosporine A (CsA) remains a well-established option for systemic treatment in moderate-to-severe psoriasis [11] due to its potent immunosuppressive action via calcineurin inhibition [12,13]. CsA forms a complex with cyclophilin A in T lymphocytes. This complex inhibits the calcium (Ca^2^⁺)/calmodulin-dependent serine/threonine phosphatase calcineurin, preventing the nuclear factor of activated T cells (NFAT) from undergoing dephosphorylation and subsequent nuclear translocation. The result is suppression of interleukin-2 (IL-2) gene transcription and reduced T-cell activation, which in turn dampens T helper 1 (Th1) and T helper 17 (Th17) immune responses that are central to psoriatic inflammation. Contemporary studies also highlight that the CsA–calcineurin axis intersects with both the mechanistic target of rapamycin complex 1 (mTORC1) and the nuclear factor kappa-light-chain-enhancer of activated B cells (NF-κB) signaling pathways, extending its influence on immune regulation [14,15,16,17].

However, its long-term use is associated with metabolic side effects, which may be aggravated by poor dietary habits or pre-existing nutritional imbalances [18,19]. While various studies have examined the role of diet in psoriasis, the majority have focused on general dietary recommendations or single nutrients. Few have comprehensively analyzed overall dietary patterns, macro- and micronutrient intake, and food frequency profiles in patients actively receiving CsA therapy [20,21,22,23].

This lack of integrated nutritional assessment in the context of systemic psoriasis treatment represents a significant knowledge gap. The pre-treatment period represents a unique therapeutic window in which nutritional status can be accurately assessed before the onset of CsA-induced metabolic alterations [6,7,22,24,25]. This timing is clinically valuable because it reflects the patient’s habitual dietary intake and baseline metabolic profile, free from drug-related confounding factors such as dyslipidemia, hypertension, or altered glucose metabolism [6,7,26]. From a research perspective, evaluating diet during this window allows for a clearer understanding of the relationship between pre-existing nutritional patterns and subsequent treatment outcomes [6,7,26]. Clinically, it provides an opportunity to implement targeted dietary interventions before therapy begins, potentially enhancing CsA efficacy, improving tolerance, and reducing the risk of adverse metabolic effects during treatment [6,7,26].

Emerging evidence also suggests that nutritional status may influence not only systemic inflammation but also the pharmacological response to CsA itself. CsA is absorbed in the intestine and extensively metabolized via CYP3A4 and P-glycoprotein, both of which are modulated by gut microbiota composition [6,7,21]. Diets low in fiber reduce microbial diversity and short-chain fatty acid production, impairing gut barrier integrity and potentially altering CsA bioavailability [27,28]. Conversely, adequate fiber intake promotes eubiosis, stabilizes drug absorption, and reduces systemic immune activation [24]. Similarly, insufficient intake of omega-3 fatty acids (EPA and DHA) limits the generation of specialized pro-resolving mediators, which synergize with CsA’s suppression of Th1/Th17 responses, thereby potentially attenuating therapeutic benefit [29]. Micronutrient deficiencies—including folate, magnesium, and zinc—may further compromise CsA efficacy by enhancing NF-κB activation, impairing antioxidant defenses, or disrupting T-cell regulation [30,31,32]. Thus, pre-treatment dietary inadequacies could contribute to variability in CsA pharmacodynamics, amplifying systemic inflammation while diminishing therapeutic responsiveness.

Therefore, the aim of the present study is to evaluate the energy value, macronutrient and micronutrient intake, and frequency of consumption of selected food groups in patients with moderate-to-severe plaque psoriasis treated with CsA. By comparing dietary intake against established dietary reference values, this study seeks to identify specific nutritional inadequacies and propose directions for dietary intervention as part of comprehensive psoriasis management.

## 2. Materials and Methods

### 2.1. Subjects

This investigation was conducted as an observational prospective cohort study. Nutritional status was assessed prior to the initiation of CsA treatment, and patients were followed for 12 weeks to evaluate clinical outcomes. This design allowed us to explore associations between baseline dietary adequacy and subsequent therapeutic response. The study initially enrolled 46 patients diagnosed with moderate-to-severe plaque psoriasis who met eligibility criteria for systemic treatment with CsA. However, due to incomplete dietary or clinical data, a full dataset was available for only 37 patients (20 men and 17 women; mean age 47.80 ± 4.87 years), who were therefore included in the final analysis. All patients received oral CsA at an average daily dose of 2.5 mg/kg body weight, administered in two divided doses, with adjustments made within the therapeutic range (2.5–5 mg/kg/day) according to clinical response and tolerability. Treatment adherence was verified at each follow-up visit, and no premature discontinuations occurred. Adverse events were limited to mild gastrointestinal discomfort and transient hypertension in a minority of patients, and none required cessation of therapy.

### 2.2. Clinical Assessment

Disease severity was evaluated using the Psoriasis Area and Severity Index (PASI) and the percentage of Body Surface Area (BSA) affected by psoriatic lesions. Assessments were performed at three planned time points: baseline (day 0, prior to CsA initiation), mid-treatment (day 42), and end of treatment (day 84). All PASI and BSA evaluations were conducted by trained dermatologists according to standardized scoring protocols.

### 2.3. Dietary Assessment

A dietary questionnaire based on 24 h dietary recall was administered to the study participants in accordance with the guidelines of the Committee on Human Nutrition Science, Polish Academy of Sciences [33]. To enhance reliability, we collected three independent 24 h recalls (two on weekdays and one on a weekend day), which reduces intra-individual variability and better captures habitual dietary intake. Portion sizes were estimated using the “Album of Photographs of Food Products and Dishes” published by the National Food and Nutrition Institute in Warsaw [34]. Nutrient content was analyzed using Dieta 6.0 software (NFNI), based on the “Tables of Composition and Nutritional Value” [35]. Anthropometric measurements were obtained and body mass index (BMI) was calculated. Dietary energy values were compared to BMI categories. The average daily energy intake and macronutrient content were determined from the 24 h recalls and compared with the applicable dietary reference values—estimated energy requirement (EER), estimated average requirement (EAR), or adequate intake (AI)—according to the dietary reference intakes established by the U.S. Institute of Medicine (National Academies of Sciences, Engineering, and Medicine) [36]. The percentage of participants meeting or failing to meet the recommendations was calculated, as well as the percentage contribution of energy from protein, fat, and carbohydrates.

### 2.4. Minerals

The dietary intake of minerals—sodium (Na), potassium (K), calcium (Ca), phosphorus (P), and magnesium (Mg)—and trace elements—iron (Fe), zinc (Zn), copper (Cu), manganese (Mn), and iodine (I)—was assessed. The results were compared to adequate intake (AI) values for Na and K and to the estimated average requirement (EAR) values for the remaining elements. For nutrients with established EAR values, the cut-point method was applied to estimate the prevalence of inadequate intake. For sodium, potassium, and manganese, the proportion of participants meeting AI was calculated. Since Polish dietary guidelines do not specify manganese intake, recommendations from the Institute of Medicine of the National Academies (USA) were used: for adults ≥ 19 years, 1.8 mg/day for women and 2.3 mg/day for men [37].

### 2.5. Vitamins

Intake of vitamins A, E, D, B_1_, B_2_, niacin, B_6_, B_12_, folate, and C was evaluated. Intake of vitamins D and E was compared to AI values, while the remaining vitamins were compared to EAR values. The prevalence of adequacy and inadequacy was calculated for each nutrient.

### 2.6. Food Frequency

In addition, a food frequency questionnaire developed by the Committee on Human Nutrition Science, Polish Academy of Sciences, covering 37 food product groups was applied [33]. Based on the reported number of days per week each product was consumed, items were classified into two categories: infrequent consumption, defined as intake once a week or less, and frequent consumption, defined as intake at least two to three times per week (for fresh and canned fish, at least once a week). Wine, vodka, and canned meat were excluded from analysis, as all participants reported rare consumption. The detailed classification of food groups, example products, and grouping criteria are presented in Supplementary Table S1. In the main analysis, food frequency categories from Supplementary Table S1 were operationalized as Group I (infrequent consumption, ≤1 time/week) and Group II (frequent consumption, ≥2–3 times/week; ≥1 time/week for fish). These groupings were then used to compare clinical outcomes (PASI, BSA) between low- and high-frequency consumers.

Accordingly, the Group I and Group II results presented below represent patients with low versus high consumption of the respective food groups, allowing for direct comparison of disease severity and therapeutic response across frequency categories.

### 2.7. Statistical Analysis

Data were analyzed using StatPlus v 1.1. (AnalystSoft Inc., Brandon, FL, USA). The Shapiro–Wilk test was used to assess normality, and sex differences were evaluated with the independent samples Student’s *t*-test. Comparisons of observed nutrient intake with established dietary reference values (EAR/AI/EER) were performed using one-sample *t*-tests. To evaluate changes in the PASI and BSA and the influence of nutrient adequacy, repeated-measures analyses were performed at baseline, day 42, and day 84.

Independent associations were assessed using linear mixed-effects regression models with the PASI or BSA as dependent variables. Each nutrient was modeled separately (adequate vs. inadequate intake). Fixed effects included time (categorical), BMI, age, sex, the baseline PASI (for PASI models) or baseline BSA (for BSA models), and baseline daily energy intake. A random intercept for each participant was included; random slopes for time were retained only when they improved model fit.

Given the number of nutrients tested across two outcomes, the Benjamini–Hochberg false discovery rate (FDR) correction (q = 0.05) was applied within each outcome family. Both raw and adjusted *p*-values are reported.

As the final analytic sample comprised 37 patients, a sensitivity analysis was performed to justify statistical power. With three repeated measures per subject and an intraclass correlation coefficient of 0.4–0.6, the effective sample size was ~55–70 observations. Under these conditions, the study had ~80% power to detect moderate standardized effects (β ≈ 0.45–0.55 SD, two-sided α = 0.05). Smaller effects may not have been detectable; the results should therefore be interpreted as exploratory. Importantly, in a previous estimation for the Polish psoriasis population, assuming ~1.2 million individuals with psoriasis, of whom ~10% have a moderate form, a 95% confidence level and 4.14% margin of error yielded a minimum required sample size of 44 patients [38]. Our cohort of 37 patients is therefore slightly below this threshold, which we acknowledge as a limitation. Two-sided *p*-values < 0.05 after FDR correction were considered statistically significant.

## 3. Results

### 3.1. Clinical Assessment

At baseline (day 0), the mean PASI score was 20.31 ± 4.16 and the mean BSA was 41.92 ± 7.35%. Mid-treatment assessment on day 42 recorded mean PASI and BSA values of 1.86 ± 1.29 and 5.95 ± 4.12%, respectively, while the final evaluation on day 84 yielded mean PASI and BSA values of 0.91 ± 0.91 and 1.87 ± 2.14%, respectively. Measurements were performed at each visit according to standardized PASI and BSA scoring protocols. To provide greater transparency, the distribution of individual PASI and BSA values across all time points is presented in Supplementary Figures S1 and S2, illustrating the variability in patient-level treatment responses.

Baseline demographic and clinical characteristics of the study cohort are summarized in Table 1. The mean age of the participants was 47.8 ± 4.9 years, with a nearly equal distribution between males (*n* = 20) and females (*n* = 17). The mean duration of psoriasis was 14.7 ± 3.4 years. At study entry, the mean baseline PASI score was 20.3 ± 4.2 and mean BSA involvement was 41.9 ± 7.4%, with no substantial sex-related differences. Most patients reported a history of topical treatment (37.8%) and phototherapy (37.8%), while systemic treatments such as methotrexate (18.9%) and acitretin (5.4%) were less common. No patients had received biological therapy prior to study enrollment. At the time of study eligibility, 40.5% were still using topical treatments, 37.8% phototherapy, 16.2% methotrexate, and 5.4% acitretin.

### 3.2. BMI Distribution

Among the 37 participants with psoriasis, underweight was observed in 8.11% of cases, normal body weight in 24.32%, overweight in 45.95%, and obesity in 21.62%. The prevalence of overweight was similar in men (45%) and women (47.06%), while obesity occurred in 20% and 23.53%, respectively (Table 2).

### 3.3. Energy and Macronutrient Intake

As shown in Table 3, both men and women had significantly lower mean daily energy intake compared with the EER (*p* < 0.05). The proportion of energy from protein exceeded the recommended 10–15% range in both sexes, whereas the percentage from fat was particularly high in men (37.8%), surpassing the upper recommended limit, and lower in women (29.89%). Carbohydrate contribution to total energy was within recommendations in women but substantially lower in men (41.9%, *p* < 0.05). Absolute protein intake met the EAR in both groups, with men showing higher mean values. Fat intake was markedly below the EAR in women and somewhat closer to recommendations in men. Intake of LA, ALA, and EPA + DHA was insufficient in both groups, with especially low mean EPA + DHA levels—well below the 250 mg/day target.

Cholesterol intake was higher in men, while dietary fiber intake met AI only in women. The mean intake of digestible carbohydrates exceeded AI in both groups, but the total carbohydrate-to-fiber ratio suggested suboptimal dietary quality.

### 3.4. Nutrient Intake Adequacy

**Figure 1:**
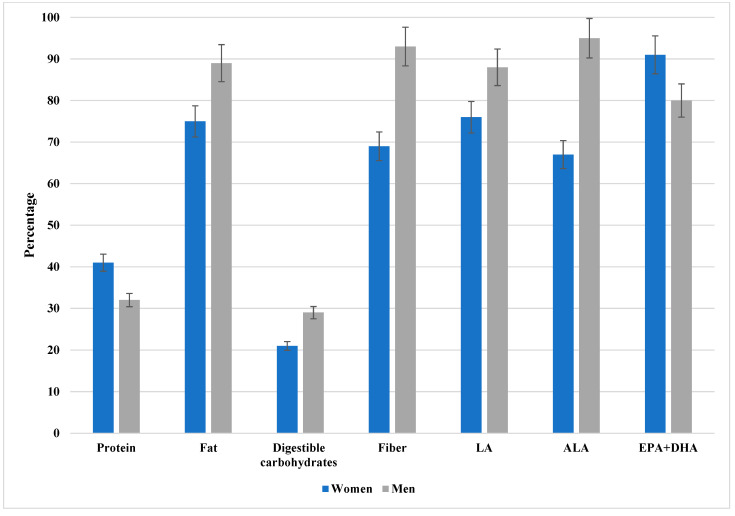
The percentage of patients with inadequate intake of protein, available carbohydrates, fat, dietary fiber, LA, ALA, and EPA + DHA. LA, linoleic acid; ALA, alpha-linoleic acid; EPA, eicosapentaenoic acid; DHA, docosahexaenoic acid. Data are presented as percentages with 95% confidence intervals (error bars) stratified by sex (women in blue, men in gray). As illustrated, a considerable proportion of patients demonstrated inadequate intake across multiple nutrients, with the most pronounced inadequacies observed for EPA + DHA. The exact percentages with corresponding confidence intervals for both women and men are provided in Supplementary Table S2.

As illustrated in Figure 1, a considerable proportion of patients demonstrated inadequate intake of protein, digestible carbohydrates, fat, dietary fiber, linoleic acid (LA), alpha-linolenic acid (ALA), and long-chain omega-3 fatty acids (EPA + DHA). The most pronounced inadequacies were observed for EPA + DHA. To improve interpretability, 95% confidence intervals for the proportions are displayed in the figure. The exact percentages with corresponding confidence intervals for both women and men are provided in Supplementary Table S2.

### 3.5. Vitamin Intake and Prevalence of Inadequacy in Patients with Psoriasis

As presented in Table 4, the mean intake of vitamin A exceeded the EAR in both sexes, although women had significantly lower values than men (*p* < 0.05). Vitamin E intake met the AI standard in women but was below AI in men. Both groups demonstrated markedly insufficient vitamin D intake, with mean values far below the AI of 15 μg/day.

Thiamine (B_1_) and riboflavin (B_2_) intake exceeded the EAR in both sexes, with significantly higher levels in men. Vitamin B_6_ intake was notably above the EAR in men across all age categories but fell below recommendations in women aged 19–50 and 51–65 years. Vitamin B_12_ intake exceeded the EAR in both sexes, with higher levels in men.

Niacin intake met the EAR in both groups without significant differences, whereas vitamin C intake was adequate in women but below the EAR in men. Folate intake was suboptimal in both sexes, averaging well below the EAR of 320 μg/day.

**Figure 2:**
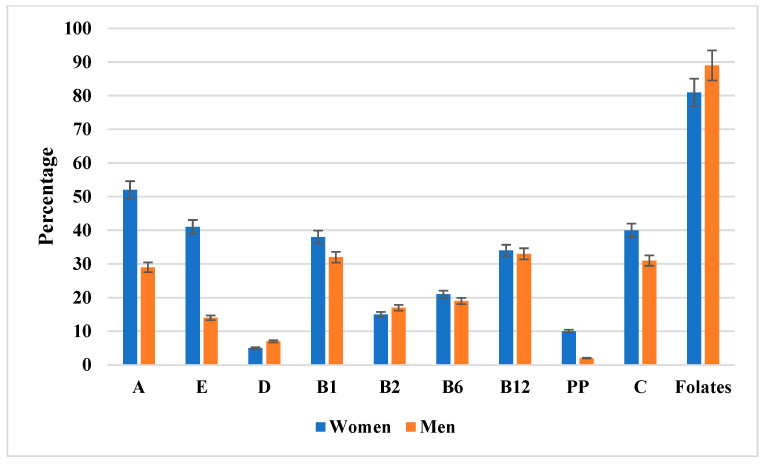
The percentage of men and women with inadequate vitamin intake relative to dietary reference values (EAR or AI). Data are presented as percentages with 95% confidence intervals (error bars) stratified by sex (women in blue, men in orange). Notably, folate intake inadequacy was the most pronounced, affecting the majority of both women and men. Additional details, including exact percentages with 95% confidence intervals, are provided in Supplementary Table S3.

Figure 2 illustrates the proportion of patients with inadequate intake of individual vitamins relative to the respective dietary reference values (EAR or AI). The most pronounced inadequacy was observed for vitamin D, with more than 90% of participants of both sexes failing to meet AI. Folate inadequacy affected approximately two-thirds of women and more than half of men. Suboptimal intake of vitamin C was prevalent among men, whereas most women achieved the EAR. In contrast, inadequate intake of A, E, and B-group vitamins was less common, with men demonstrating a higher adequacy rate for vitamins B_6_ and B_12_. To facilitate precise evaluation, the detailed percentages with 95% confidence intervals are presented in Supplementary Table S3.

### 3.6. Mineral Component Intake and Prevalence of Inadequacy in Patients with Psoriasis

As shown in Table 5, Na and K intake exceeded AI in both sexes, with significantly higher mean Na levels in men (*p* < 0.05). Ca intake was below the EAR across all age groups, with particularly low values in women aged 51–65 years. P intake substantially exceeded the EAR in both sexes.

Mg intake met the EAR in most subgroups, although women aged 19–50 years and men aged 51–65 years were closer to the lower threshold. Zn, Cu, and Mn intake exceeded dietary reference values in both sexes, while Fe intake met or exceeded the EAR, with significantly higher values in men. I intake was below the EAR in women but exceeded the requirement in men.

**Figure 3:**
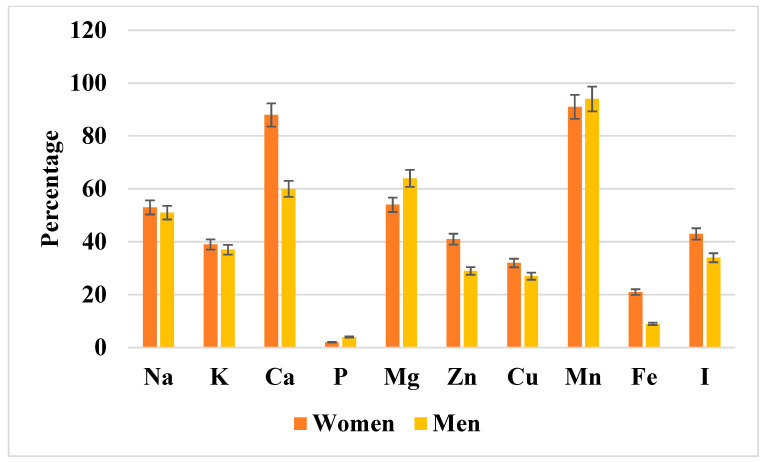
The percentage of men and women with inadequate mineral component intake relative to dietary reference values (EAR or AI). Data are presented as percentages with 95% confidence intervals (error bars) stratified by sex (women in orange, men in yellow). As shown, marked inadequacies were observed for calcium, magnesium, manganese, and iodine, particularly among women. The exact percentages with corresponding confidence intervals for both sexes are provided in Supplementary Table S4. Na, sodium; K, potassium; Ca, calcium; P, phosphorus; Mg, magnesium; Zn, zinc; Cu, copper; Mn, manganese; Fe, iron; I, iodine.

Figure 3 illustrates the prevalence of inadequate mineral intake relative to dietary reference values (EAR or AI). The highest inadequacy rates were observed for calcium (in both sexes, particularly among older women) and iodine (notably in women). Magnesium inadequacy was less common but still affected certain subgroups, whereas sodium, potassium, phosphorus, and trace elements such as zinc and manganese showed the lowest prevalence of inadequacy. For detailed percentages with corresponding 95% confidence intervals, see Supplementary Table S4.

### 3.7. Comparison of PASI and BSA Changes According to Baseline Nutrient Intake Adequacy

Analysis of PASI (Table 6) and BSA (Table 7) dynamics demonstrated that patients with adequate intake of key nutrients generally exhibited lower baseline disease severity and achieved greater reductions during treatment compared to those with inadequate intake. The most pronounced differences were observed for fiber, EPA + DHA, vitamins A, E, and D, folate, magnesium, and zinc, where both PASI and BSA values were significantly lower across follow-up points (*p* < 0.05). For other dietary components, between-group differences were minimal and did not reach statistical significance (*p* > 0.05), suggesting a limited impact on treatment outcomes.

### 3.8. Impact of Nutrient Deficiency and Clinical Covariates on Disease Severity Dynamics (PASI and BSA) During Cyclosporine A Therapy

Mixed-model analyses revealed that nutrient inadequacy was significantly associated with higher PASI and BSA values at baseline and, for several nutrients, at subsequent follow-up points (Table 8).

For BSA, large baseline differences were observed for EPA + DHA (β = 10.37, 95% CI [5.26, 15.47], *p* = 0.0001, q = 0.0006), fiber (β = 10.53, 95% CI [5.77, 15.29], *p* < 0.001, q = 0.0003), vitamin A (β = 9.16, 95% CI [4.58, 13.75], *p* = 0.0001, q = 0.0006), and vitamin D (β = 11.97, 95% CI [4.12, 19.83], *p* = 0.0028, q = 0.0138). These nutrients also showed sustained effects on day 42, with fiber and vitamin A remaining significant after FDR adjustment. By day 84, most associations attenuated, although fiber and EPA + DHA still exhibited modest differences near significance thresholds.

For the PASI, deficits in fiber, vitamin A, vitamin D, and EPA + DHA were associated with significantly higher baseline scores. Fiber inadequacy showed the strongest effects (β = 6.04, 95% CI [3.78, 8.29], *p* < 0.001, q < 0.001). On day 42, vitamin A remained robustly associated (β = 2.24, 95% CI [1.06, 3.42], *p* = 0.0002, q = 0.0011), and by day 84 the vitamin A effect persisted (β = 2.71, 95% CI [1.40, 4.01], *p* < 0.001, q = 0.0003). Other nutrients (folate, magnesium, zinc) did not show consistent or significant associations after FDR correction.

Overall, nutrient inadequacy—especially in fiber, vitamin A, vitamin D, and EPA + DHA—was linked to worse psoriasis severity, with the strongest and most consistent effects seen for fiber and vitamin A across multiple time points (Table 8).

Covariate estimates are presented in Supplementary Table S5. As expected, higher baseline PASI and BSA values strongly predicted follow-up severity, while age, sex, BMI, and baseline energy intake showed no consistent associations across nutrient models.

## 4. Discussion

This study provides a detailed evaluation of dietary intake, nutrient adequacy, and habitual food consumption in patients with moderate-to-severe plaque psoriasis prior to CsA therapy. Assessing nutrition before systemic immunosuppression offers an unaltered view of baseline dietary patterns, free from CsA-related metabolic effects, and enables accurate appraisal of pre-treatment nutritional status. To our knowledge, few studies have examined this specific therapeutic window, despite its potential importance for optimizing treatment strategies and supporting long-term disease control [25].

Recent systematic reviews and randomized controlled trials further support the immunomodulatory effects of specific nutrients in psoriasis. For example, Formisano et al. found significantly lower serum 25-hydroxyvitamin D levels in psoriasis patients vs. controls, though the impact of oral vitamin D supplementation on PASI was modest in meta-analysis [39]. Similarly, Dai et al. in PLOS ONE reviewed RCTs and highlighted safety but little overall PASI reduction with supplementation, with subgroup effects according to vitamin D form or region [40]. Meanwhile, ω-3 fatty acid supplementation, including herring roe oil, has shown favorable modulations of inflammatory cytokines and immune cell phenotypes [41]. Incorporating such evidence strengthens the biological plausibility of our findings.

Even before systemic treatment, patients displayed marked qualitative and quantitative dietary imbalances. Excess total energy and saturated fatty acid (SFA) intake, combined with low consumption of polyunsaturated fatty acids (PUFAs) and dietary fiber, were accompanied by suboptimal intakes of multiple essential micronutrients. These patterns align with previous reports linking psoriasis to pro-inflammatory diets rich in saturated fats, refined carbohydrates, and processed foods, but poor in fiber, omega-3 fatty acids, fruits, and vegetables—profiles associated with heightened systemic inflammation and greater disease severity [6,21]. Mechanistically, SFA-rich diets activate Toll-like receptor 4 (TLR4) signaling, triggering NF-κB-mediated transcription of cytokines such as TNF-α, IL-6, and IL-1β [30,31,32]. Inadequate omega-3 PUFA intake limits synthesis of specialized pro-resolving mediators (resolvins, protectins, maresins) [29,42], while low dietary fiber reduces gut microbiota-derived short-chain fatty acids (SCFAs), impairing intestinal barrier integrity and immune regulation [27,28].

Food frequency data confirmed a predominantly Western-type diet—low in vegetables, fruit, and whole grains, but high in processed meats, refined carbohydrates, and sugar-sweetened products [43,44,45,46]—known to perpetuate low-grade inflammation and worsen psoriasis severity [47,48]. In contrast, Mediterranean-style diets rich in plant foods, omega-3-rich fish, and olive oil are associated with reduced disease activity and improved quality of life [49,50].

Macronutrient analysis revealed that, although mean energy intake was below estimated requirements in both sexes, macronutrient distribution was imbalanced. Protein accounted for an excessive proportion of energy—mainly from animal sources—with elevated cholesterol intake. Men consumed excess total fat with insufficient carbohydrates, while women had higher total carbohydrate intake, but with an overrepresentation of rapidly digestible carbohydrates. Notably, intakes of LA, ALA, and especially EPA + DHA were well below adequacy thresholds. This unfavorable lipid profile—high in SFAs and cholesterol but low in PUFAs—promotes a pro-inflammatory metabolic environment and limits endogenous inflammation-resolving capacity [51,52].

Carbohydrate quality also suggested inflammatory potential [53]: women consumed excessive digestible carbohydrates, while men had reduced total carbohydrate intake alongside a ~40% fiber deficit. High refined carbohydrate intake and low fiber intake reduce gut microbial diversity, diminish SCFA production, and promote systemic inflammation [54,55,56]. Micronutrient inadequacies were common, particularly in vitamins C, E, A, β-carotene, and D. Low vitamin C and E intake impairs antioxidant defense [57], vitamin A deficiency affects keratinocyte differentiation, and β-carotene provides both antioxidant and provitamin A activity [58]. Vitamin D deficiency—common in psoriasis—contributes to immune dysregulation, favoring Th1/Th17 activation and reduced Treg function, which may impair therapy responsiveness [59,60].

B-group vitamin deficiencies (B6, B12, folate) are clinically relevant through their role in homocysteine metabolism [61,62,63], with hyperhomocysteinemia linked to endothelial dysfunction and cardiovascular risk in psoriasis [64,65]. Zn deficiency may impair epidermal regeneration and immune modulation, magnesium insufficiency may enhance NF-κB and NLRP3 inflammasome activation [66,67], and iron imbalances affect mitochondrial function, erythropoiesis, and immunity [68]. Calcium and phosphorus imbalances can impair keratinocyte differentiation [69,70], while high sodium and low potassium may raise blood pressure and promote Th17 differentiation—effects potentially exacerbating CsA-induced hypertension [71,72,73].

Disease severity analysis showed that patients with specific nutrient deficiencies had higher PASI and BSA values at baseline and maintained a relative disadvantage during therapy [8]. Although PASI and BSA values declined in all patients receiving CsA, improvement was attenuated in those with dietary inadequacies [23,74,75]. Multivariable analysis confirmed nutrient adequacy as an independent predictor of lower PASI and BSA values, suggesting a direct influence on the rate of clinical improvement. Higher BMI, older age, and male sex were associated with less favorable outcomes [2,76]. While treatment duration was the strongest predictor of improvement, adequate nutritional status conferred an additive benefit beyond CsA’s pharmacologic effects. Although several between-group differences in the PASI and BSA reached statistical significance, it is equally important to consider their clinical relevance. In psoriasis, a reduction of ~3–5 PASI points or achieving PASI 75 is often regarded as the minimal clinically important difference (MCID). Most patients in both adequate and inadequate nutrient groups achieved reductions far exceeding these thresholds, indicating that the observed differences, while statistically robust, may represent relative rather than absolute clinical benefit.

Clinically, these findings highlight the value of nutritional assessment before systemic therapy. Early dietary modifications may help reduce systemic inflammation, optimize baseline metabolic status, and potentially mitigate CsA-related metabolic side effects. Integrating nutritional optimization into psoriasis management could improve adherence to lifestyle interventions and sustain long-term therapeutic benefits [77,78,79].

Growing evidence supports diet as an important adjunct in psoriasis management. Dietary interventions such as calorie restriction, weight reduction, and adherence to Mediterranean-style diets have been associated with lower disease severity and improved treatment responses, particularly when combined with systemic therapies [22]. Nutritional strategies that emphasize balanced macronutrient intake and anti-inflammatory food sources may therefore represent an accessible, non-pharmacological complement to pharmacotherapy [80].

Beyond macronutrient balance, antioxidant-rich diets—providing vitamins C and E, carotenoids, polyphenols, and omega-3 fatty acids—have been shown to reduce oxidative stress and inflammatory cytokine release, both of which are strongly implicated in psoriatic pathogenesis [24]. It should be noted, however, that while nutrients such as dietary fiber and omega-3 fatty acids are well recognized to influence gut microbiota composition and systemic inflammation, direct mechanistic evidence linking these pathways to CsA pharmacodynamics remains limited. Therefore, our findings should be interpreted as demonstrating associations between pre-treatment nutritional adequacy and treatment response, rather than establishing causality.

Likewise, psychological stress is increasingly recognized as a trigger and exacerbating factor of psoriasis [81]. Stress and depression may activate neuroendocrine and immune pathways, intensifying Th1/Th17-driven inflammation [82,83]. Addressing these aspects through stress management strategies, mindfulness, and psychosocial support could therefore improve both clinical and quality-of-life outcomes [81].

It is important to highlight that the impact of dietary and lifestyle interventions may not be uniform across all patients. Individuals with a strong genetic predisposition (e.g., HLA-C*06:02 or polymorphisms affecting one-carbon metabolism) may experience heightened inflammatory responses and oxidative stress. In these patients, targeted dietary strategies—such as folate and B-group vitamin optimization or enhanced antioxidant intake—could be particularly beneficial. Conversely, in patients without a strong genetic background, lifestyle-related triggers such as obesity, smoking, alcohol use, and stress may play a proportionally larger role, and nutritional interventions might exert a more pronounced clinical effect. This gene–environment interplay highlights the need for individualized dietary counseling integrated into comprehensive psoriasis care [84,85,86].

Limitations include the self-reported nature of dietary data, risk of recall bias, cross-sectional design, and absence of a psoriasis-free control group. A key limitation of our study is that dietary intake was assessed using 24 h dietary recalls rather than biochemical nutrient measurements, which may introduce recall bias and estimation error; although we used three independent recalls (including weekdays and a weekend day) to improve reliability, future studies should incorporate biomarker-based assessment and prospective dietary interventions. Although we used three independent 24 h recalls (including weekdays and a weekend day) in combination with an FFQ to improve reliability, these methods remain dependent on participant memory and estimation of portion sizes. Recall bias and day-to-day variability are therefore unavoidable, and future studies should complement questionnaire data with biomarker-based nutritional assessment. We acknowledge this as a primary limitation of the present study. Additionally, although 46 patients were initially recruited, only 37 provided complete dietary and clinical datasets suitable for longitudinal analysis. This number is slightly below the minimum of 44 participants estimated in a previous study of the Polish psoriasis population, which assumed ~1.2 million individuals with psoriasis, ~10% with a moderate form, a 95% confidence level, and a 4.14% margin of error [38]. While this shortfall may have reduced statistical power for smaller effects, the repeated-measures design with three assessments per subject increased the effective sample size to approximately 55–70 observations, providing sufficient power to detect moderate effects. Nevertheless, our findings should be interpreted with caution and regarded as exploratory and hypothesis-generating.

Because a large number of nutrients were tested across two outcomes, the risk of false positive findings needed to be addressed. To minimize this, we applied the Benjamini–Hochberg false discovery rate (FDR) correction within each outcome family and reported both raw and adjusted *p*-values. Nevertheless, the exploratory nature of these analyses should be emphasized, and the results require replication in larger cohorts. Another limitation is the dichotomization of nutrient intake into ‘adequate’ versus ‘inadequate’ categories, which may oversimplify the inherently continuous nature of dietary variables; although sensitivity analyses with continuous values yielded consistent results, this simplification should be taken into account when interpreting the findings

Future work should complement self-reported dietary intake with objective biomarkers such as circulating fatty acids, vitamins, and trace minerals, which could improve precision and validate dietary estimates.

## 5. Conclusions

Patients with moderate-to-severe plaque psoriasis displayed substantial dietary imbalances before initiating CsA, including excess saturated fat, insufficient polyunsaturated fatty acids and fiber, and widespread micronutrient deficiencies. These nutritional inadequacies were associated with higher disease severity at baseline and persisted as independent predictors of poorer clinical outcomes during treatment, even after accounting for BMI, age, sex, and therapy duration. Optimizing dietary intake prior to systemic immunosuppression may accelerate therapeutic response, enhance overall treatment efficacy, and support long-term disease control. Integrating nutritional assessment and targeted dietary intervention into psoriasis management protocols represents a feasible and potentially impactful strategy to improve patient outcomes.

## Figures and Tables

**Table 1:** Baseline demographic and clinical characteristics of study participants.

Variable		Total (*n* = 37)	Male (*n* = 20)	Female (*n* = 17)
Age, years (mean ± SD)		47.80 ± 4.87	48.2 ± 5.1	47.3 ± 4.6
Psoriasis duration, years (mean ± SD)		14.65 ± 3.42	15.0 ± 3.6	14.3 ± 3.2
PASI score, baseline (mean ± SD)		20.31 ± 4.16	20.5 ± 4.4	20.1 ± 4.0
BSA %, baseline (mean ± SD)		41.92 ± 7.35%	42.3 ± 7.6	41.5 ± 7.2
Treatment of psoriasis used in the past before the study (more than 1 response possible)	Topical treatment	14 (37.85)	8 (40%)	6 (35.3%)
	Methotrexate	7 (18.90%)	4 (20%)	3 (17.6%)
	Acitretin	2 (5.40%)	1 (5%)	1 (5.9%)
	Light therapy (UVB 311)	14 (37.80%)	8 (40%)	6 (35.3%)
	Biological treatment	0	0	0
Psoriasis treatment used at the time of study eligibility (more than 1 response possible)	Topical treatment	15 (40.50%)	9 (45%)	6 (35.3%)
	Methotrexate	6 (16.20%)	3 (15%)	3 (17.6%)
	Acitretin	2 (5.40%)	1 (5%)	1 (5.9%)
	Light therapy (UVB 311)	14 (37.80%)	8 (40%)	6 (35.3%)
	Biological treatment	0	0	0

PASI, Psoriasis Area and Severity Index; BSA, Body Surface Area. Data are presented as mean ± standard deviation (SD) for continuous variables and as number (percentage) for categorical variables.

**Table 2:** Distribution of BMI in individuals with psoriasis.

Body Mass Index (BMI)	Number (Percentage) of Patients		
	Men (*n* = 20)	Women (*n* = 17)	General (*n* = 37)
Underweight (<18.5 kg/m^2^)	1 (5%)	2 (11.76%)	3 (8.11%)
Normal body weight (18.5–24.9 kg/m^2^)	6 (30%)	3 (17.65%)	9 (24.32%)
Overweight (25–29.9 kg/m^2^)	9 (45%)	8 (47.06%)	17 (45.95%)
Obesity (>30.0 kg/m^2^)	4 (20%)	4 (23.53%)	8 (21.62%)

BMI, body mass index.

**Table 3:** Estimated energy requirements, estimated average requirements, and adequate intake standards for energy and nutrients in men and women.

Energy and Nutrients	Gender	Mean ± SD	Norm		*p*-Value Student’s *t*-Test
			Type of Standard	Quantity/Day	
Energy [kcal]	Women (*n* = 17)	1765.19 ± 109.23	EER	2290.32 ± 189.76	<0.05
	Men (*n* = 20)	2345.56 ± 376.23		3090.40 ± 286.87	
% energy from protein	Women (*n* = 17)	18.90 ± 5.56	-	10–15	>0.05
	Men (*n* = 20)	21.30 ± 5.50			
% energy from fat	Women (*n* = 17)	29.89 ± 7.76	-	20–35	<0.05
	Men (*n* = 20)	37.80 ± 10.10			
% energy from carbohydrates	Women (*n* = 17)	53.19 ± 8.76	-	50–70	<0.05
	Men (*n* = 20)	41.90 ± 9.80			
Protein [g]	Women (*n* = 17)	56.81 ± 9.12	EAR	54.90 ± 4.10	>0.05
	Men (*n* = 20)	61.87 ± 8.76		70.10 ± 9.70	
Animal protein [g]	Women (*n* = 17)	38.90 ± 5.50	-	-	<0.05
	Men (*n* = 20)	67.80 ± 10.10			
Plant protein [g]	Women (*n* = 17)	17.91 ± 4.40	-	-	>0.05
	Men (*n* = 20)	18.82 ± 7.65			
Fat [g]	Women (*n* = 17)	51.12 ± 9.12	EAR	76.20 ± 8.90	<0.05
	Men (*n* = 20)	71.87 ± 10.18		109.90 ± 21.80	
LA [g]	Women (*n* = 17)	4.90 ± 1.90	AI	10.20 ± 0.90	>0.05
	Men (*n* = 20)	6.70 ± 2.90		15.20 ± 5.60	
ALA [g]	Women (*n* = 17)	0.90 ± 0.70		1.20 ± 0.10	>0.05
	Men (*n* = 20)	0.80 ± 0.40		1.90 ± 0.50	
EPA + DHA [mg]	Women (*n* = 17)	87.12 ± 56.90		250	<0.05
	Men (*n* = 20)	145.98 ± 56.87			
Cholesterol [g]	Women (*n* = 17)	200.20 ± 96.80	-	-	<0.05
	Men (*n* = 20)	289.98 ± 109.87			
Unsaturated fatty acids [g]	Women (*n* = 17)	20.40 ± 10.80	-	-	>0.05
	Men (*n* = 20)	22.90 ± 7.60			
Monounsaturated fatty acids [g]	Women (*n* = 17)	20.10 ± 8.10	-	-	<0.05
	Men (*n* = 20)	25.10 ± 7.87			
Polyunsaturated fatty acids [g]	Women (*n* = 17)	7.80 ± 6.90	-	-	>0.05
	Men (*n* = 20)	9.00 ± 1.80			
Carbohydrates [g]	Women (*n* = 17)	250.30 ± 109.80	-	-	<0.05
	Men (*n* = 20)	200.10 ± 90.70			
Digestible carbohydrates [g]	Women (*n* = 17)	223.45 ± 96.10	AI	100	<0.05
	Men (*n* = 20)	187.60 ± 67.80			
Fiber [g]	Women (*n* = 17)	21.93 ± 8.24	AI	25	<0.05
	Men (*n* = 20)	14.80 ± 6.10			

Data are presented as mean ± standard deviation (SD). Group sizes: women (*n* = 17); men (*n* = 20). Standards are reported as estimated energy requirements (EERs), estimated average requirements (EAR), or adequate intake (AI), where applicable. Comparisons between sexes were conducted using independent-samples Student’s *t*-test. Data distribution was verified using Shapiro–Wilk test, confirming normality assumptions. Abbreviations: LA, linoleic acid; ALA, alpha-linolenic acid; EPA, eicosapentaenoic acid; DHA, docosahexaenoic acid; EER, estimated energy requirement; EAR, estimated average requirement; AI, adequate intake; kcal, kilocalorie; g, gram; mg, milligram.

**Table 4:** Mean daily vitamin intake in men and women with psoriasis compared to dietary reference values (EAR, AI).

Vitamin	Gender	Mean ± SD	Norm			*p*-Value Student’s *t*-Test
			Type of Standard	Age	Quantity/Day	
A [μg]	Women (*n* = 17)	698.24 ± 458.19	EAR	≥19	500	<0.05
	Men (*n* = 20)	948.52 ± 390.98			630	
E [mg]	Women (*n* = 17)	9.19 ± 3.45	AI	≥19	8	<0.05
	Men (*n* = 20)	7.19 ± 2.53			10	
D [μg]	Women (*n* = 17)	1.87 ± 0.98	AI	≥19	15	<0.05
	Men (*n* = 20)	3.79 ± 1.91			15	
B_1_ [mg]	Women (*n* = 17)	1.09 ± 0.87	EAR	≥19	0.9	<0.05
	Men (*n* = 20)	1.32 ± 0.51			1.1	
B_2_ [mg]	Women (*n* = 17)	1.23 ± 0.81	EAR	≥19	0.9	<0.05
	Men (*n* = 20)	1.56 ± 0.65			1.1	
B_6_ [mg]	Women (*n* = 17)	0.81 ± 0.12	EAR	19–50	1.1	<0.05
	Men (*n* = 20)	1.98 ± 0.22			1.1	
	Women (*n* = 17)	0.98 ± 0.65		51–65	1.3	<0.05
	Men (*n* = 20)	1.78 ± 0.49			1.4	
B_12_ [μg]	Women (*n* = 17)	2.76 ± 0.81	EAR	≥19	2.0	<0.05
	Men (*n* = 20)	3.98 ± 0.19			2.0	
Niacin [mg]	Women (*n* = 17)	20.98 ± 4.67	EAR	≥19	11	>0.05
	Men (*n* = 20)	22.56 ± 2.91			12	
C [mg]	Women (*n* = 17)	70.09 ± 34.12	EAR	≥19	60	<0.05
	Men (*n* = 20)	56.90 ± 10.87			75	
Folate [μg]	Women (*n* = 17)	190.89 ± 87.90	EAR	≥19	320	>0.05
	Men (*n* = 20)	209.19 ± 45.89			320	

Data are presented as the mean ± standard deviation. Group sizes: men (*n* = 20); women (*n* = 17). Data distribution was tested using the Shapiro–Wilk test; all variables met normality assumptions, and comparisons between sexes were performed with the independent-samples Student’s *t*-test.

**Table 5:** Mean daily mineral component intake in men and women with psoriasis compared to dietary reference values (EAR, AI).

Mineral Component	Gender	Mean ± SD	Norm			*p*-Value Student’s *t*-Test
			Type of Standard	Age	Quantity/Day	
Na [mg/day]	Women (*n* = 17)	2789.98 ± 1456.89	AI	≥19	1500	<0.05
	Men (*n* = 20)	3450.80 ± 1789.19			1500	
K [mg/day]	Women (*n* = 17)	3671.08 ± 1567.90	AI	≥19	3500	<0.05
	Men (*n* = 20)	3210.91 ± 1789.87			2500	
Ca [mg]	Women (*n* = 17)	509.98 ± 108.76	EAR	19–50	800	<0.05
	Men (*n* = 20)	598.40 ± 234.76			1000	
	Women (*n* = 17)	456.98 ± 90.76		51–65	800	<0.05
	Men (*n* = 20)	578.10 ± 210.91			1000	<0.05
P [mg/day]	Women (*n* = 17)	1345.67 ± 451.09	EAR	≥19	580	>0.05
	Men (*n* = 20)	1450.56 ± 213.89			580	
Mg [mg/day]	Women (*n* = 17)	256.87 ± 45.56	EAR	19–50	255	<0.05
	Men (*n* = 20)	320.90 ± 89.19			330	
	Women (*n* = 17)	276.54 ± 51.80		51–65	265	>0.05
	Men (*n* = 20)	312.22 ± 70.10			350	
Zn [mg/day]	Women (*n* = 17)	8.90 ± 1.98	EAR	≥19	6.80	>0.05
	Men (*n* = 20)	10.80 ± 210			9.40	
Cu [mg/day]	Women (*n* = 17)	0.90 ± 0.21	EAR	≥19	0.70	>0.05
	Men (*n* = 20)	0.95 ± 0.19			0.70	
Mn [mg/day]	Women (*n* = 17)	3.80 ± 1.09	AI	≥19	1.80	>0.05
	Men (*n* = 20)	4.50 ± 1.01			2.30	
Fe [mg/day]	Women (*n* = 17)	8.90 ± 2.18	EAR	≥19	8	<0.05
	Men (*n* = 20)	12.80 ± 5.70			6	
I [μg/day]	Women (*n* = 17)	90.87 ± 34.15	EAR	≥19	95	<0.05
	Men (*n* = 20)	149.87 ± 41.98			95	

Data are presented as the mean ± standard deviation. Group sizes: men (*n* = 20); women (*n* = 17). Data distribution was tested using the Shapiro–Wilk test; all variables met normality assumptions, and comparisons between sexes were performed with the independent-samples Student’s *t*-test. Na, sodium; K, potassium; Ca, calcium; P, phosphorus; Mg, magnesium; Zn, zinc; Cu, copper; Mn, manganese; Fe, iron; I, iodine.

**Table 6:** Changes in PASI depending on adequate vs. inadequate intake of dietary components.

Dietary Component	Intake	0 Day	42 Days	84 Days
Protein	Adequate	19.99 ± 4.1	1.73 ± 1.2	0.85 ± 0.85
	Inadequate	20.49 ± 4.2	1.93 ± 1.34	0.95 ± 0.95
Fat	Adequate	20.22 ± 4.14	1.82 ± 1.26	0.9 ± 0.89
	Inadequate	20.72 ± 4.25	2.02 ± 1.4	1.0 ± 0.99
Digestible carbohydrates	Adequate	19.94 ± 4.09	1.71 ± 1.18	0.84 ± 0.84
	Inadequate	20.44 ± 4.19	1.91 ± 1.32	0.94 ± 0.93
Fiber *	Adequate	19.55 ± 4.01	1.67 ± 1.16	0.82 ± 0.82
	Inadequate	23.55 ± 4.83	2.67 ± 1.85	1.32 ± 1.31
LA	Adequate	20.22 ± 4.14	1.82 ± 1.26	0.9 ± 0.89
	Inadequate	20.72 ± 4.25	2.02 ± 1.4	1.0 ± 0.99
ALA	Adequate	20.22 ± 4.14	1.82 ± 1.26	0.89 ± 0.89
	Inadequate	20.72 ± 4.25	2.02 ± 1.4	0.99 ± 0.99
EPA + DHA *	Adequate	19.71 ± 4.04	1.71 ± 1.18	0.84 ± 0.84
	Inadequate	23.71 ± 4.86	2.71 ± 1.88	1.34 ± 1.33
Vit. A *	Adequate	17.95 ± 3.68	1.27 ± 0.88	0.62 ± 0.62
	Inadequate	21.95 ± 4.5	2.27 ± 1.57	0.62 ± 0.62
Vit. E *	Adequate	17.43 ± 3.57	1.14 ± 0.79	0.55 ± 0.55
	Inadequate	21.43 ± 4.39	2.14 ± 1.48	1.05 ± 1.05
Vit. D *	Adequate	16.55 ± 3.39	0.92 ± 0.64	0.44 ± 0.44
	Inadequate	20.55 ± 4.21	1.92 ± 1.33	0.94 ± 0.94
Vit B_1_	Adequate	19.99 ± 4.1	1.73 ± 1.2	0.85 ± 0.85
	Inadequate	20.49 ± 4.2	1.93 ± 1.34	0.95 ± 0.94
Vit. B_6_	Adequate	19.91 ± 4.08	1.7 ± 1.18	0.83 ± 0.83
	Inadequate	20.41 ± 4.18	1.9 ± 1.32	0.93 ± 0.93
Vit. B_12_	Adequate	19.98 ± 4.1	1.72 ± 1.2	0.85 ± 0.84
	Inadequate	20.48 ± 4.2	1.92 ± 1.33	0.95 ± 0.94
Niacin	Adequate	19.84 ± 4.07	1.67 ± 1.16	0.82 ± 0.82
	Inadequate	20.34 ± 4.17	1.87 ± 1.3	0.92 ± 0.92
Vit. C	Adequate	19.99 ± 4.1	1.73 ± 1.2	0.85 ± 0.85
	Inadequate	20.49 ± 4.2	1.93 ± 1.34	0.95 ± 0.95
Folate *	Adequate	19.71 ± 4.04	1.71 ± 1.18	0.84 ± 0.84
	Inadequate	23.71 ± 4.86	2.71 ± 1.88	1.34 ± 1.33
Na	Adequate	20.07 ± 4.11	1.76 ± 1.22	0.87 ± 0.86
	Inadequate	20.57 ± 4.22	1.96 ± 1.36	0.97 ± 0.96
K	Adequate	20.0 ± 4.1	1.73 ± 1.2	0.85 ± 0.85
	Inadequate	20.5 ± 4.2	1.93 ± 1.34	0.95 ± 0.95
Ca	Adequate	20.18 ± 4.14	1.8 ± 1.25	1.8 ± 1.25
	Inadequate	20.68 ± 4.24	2.0 ± 1.39	2.0 ± 1.39
P	Adequate	19.83 ± 4.06	1.66 ± 1.15	0.82 ± 0.81
	Inadequate	20.33 ± 4.17	1.86 ± 1.29	0.92 ± 0.91
Mg *	Adequate	18.67 ± 3.83	1.45 ± 1.0	0.71 ± 0.71
	Inadequate	22.67 ± 4.65	2.45 ± 1.7	1.21 ± 1.2
Zn *	Adequate	17.71 ± 3.63	1.21 ± 0.84	0.59 ± 0.59
	Inadequate	21.71 ± 4.45	2.21 ± 1.53	1.09 ± 1.08
Cu	Adequate	19.96 ± 4.09	1.72 ± 1.19	0.84 ± 0.84
	Inadequate	20.46 ± 4.19	1.92 ± 1.33	0.94 ± 0.94
Mn	Adequate	20.27 ± 4.16	1.84 ± 1.28	0.91 ± 0.9
	Inadequate	20.77 ± 4.26	2.04 ± 1.42	1.01 ± 1.0
Fe	Adequate	19.89 ± 4.08	1.69 ± 1.17	0.83 ± 0.83
	Inadequate	20.39 ± 4.18	1.89 ± 1.31	0.93 ± 0.92
I	Adequate	20.0 ± 4.1	1.73 ± 1.2	0.85 ± 0.85
	Inadequate	20.5 ± 4.2	1.93 ± 1.34	0.95 ± 0.95

Values are presented as the mean ± standard deviation (SD) for the PASI at baseline (day 0), day 42, and day 84. Groups were classified according to adequate or inadequate intake of each dietary component prior to treatment initiation. An asterisk indicates a statistically significant difference between groups at the given time point (*p* < 0.05; Student’s *t*-test). LA, linoleic acid; ALA, alpha-linoleic acid; EPA, eicosapentaenoic acid; DHA, docosahexaenoic acid; Na, sodium; K, potassium; Ca, calcium; P, phosphorus; Mg, magnesium; Zn, zinc; Cu, copper; Mn, manganese; Fe, iron; I, iodine.

**Table 7:** Changes in BSA depending on adequate vs. inadequate intake of dietary components.

	Intake	0 Day	42 Days	84 Days
Protein	Adequate	41.29 ± 7.24	5.7 ± 3.95	1.74 ± 1.99
	Inadequate	41.74 ± 7.32	5.88 ± 4.07	1.83 ± 2.1
Fat	Adequate	42.74 ± 7.49	6.28 ± 4.35	2.03 ± 2.32
	Inadequate	20.72 ± 4.25	2.02 ± 1.4	1.0 ± 0.99
Digestible carbohydrates	Adequate	41.17 ± 7.22	5.65 ± 3.92	1.72 ± 1.96
	Inadequate	42.17 ± 7.39	6.05 ± 4.19	1.92 ± 2.19
Fiber *	Adequate	40.4 ± 7.08	5.57 ± 3.86	1.68 ± 1.92
	Inadequate	48.4 ± 8.49	7.57 ± 5.25	2.68 ± 3.06
LA	Adequate	41.74 ± 7.32	5.88 ± 4.07	1.83 ± 2.1
	Inadequate	42.74 ± 7.49	6.28 ± 4.35	2.03 ± 2.32
ALA	Adequate	41.73 ± 7.32	5.88 ± 4.07	1.83 ± 2.09
	Inadequate	42.73 ± 7.49	6.28 ± 4.35	2.03 ± 2.32
EPA + DHA *	Adequate	40.72 ± 7.14	5.65 ± 3.92	1.72 ± 1.96
	Inadequate	48.72 ± 8.54	7.65 ± 5.3	2.72 ± 3.11
Vit. A *	Adequate	37.2 ± 6.52	4.77 ± 3.31	1.28 ± 1.46
	Inadequate	45.2 ± 7.92	6.77 ± 4.69	2.28 ± 2.61
Vit. E *	Adequate	36.16 ± 6.34	4.51 ± 3.13	1.15 ± 1.31
	Inadequate	44.16 ± 7.74	6.51 ± 4.51	2.15 ± 2.46
Vit. D *	Adequate	34.4 ± 6.03	4.07 ± 2.82	0.93 ± 1.06
	Inadequate	42.4 ± 7.43	6.07 ± 4.21	1.93 ± 2.21
Vit B_1_	Adequate	41.27 ± 7.24	5.69 ± 3.94	1.74 ± 1.99
	Inadequate	42.27 ± 7.41	6.09 ± 4.22	1.94 ± 2.22
Vit. B_6_	Adequate	41.12 ± 7.21	5.63 ± 3.9	1.71 ± 1.95
	Inadequate	42.12 ± 7.38	6.03 ± 4.18	1.91 ± 2.18
Vit. B_12_	Adequate	41.26 ± 7.23	5.69 ± 3.94	1.73 ± 1.98
	Inadequate	42.26 ± 7.41	6.09 ± 4.22	1.93 ± 2.21
Niacin	Adequate	40.98 ± 7.18	5.58 ± 3.86	1.68 ± 1.92
	Inadequate	41.98 ± 7.36	5.98 ± 4.14	1.88 ± 2.15
Vit. C	Adequate	41.28 ± 7.24	5.7 ± 3.94	1.74 ± 1.99
	Inadequate	42.28 ± 7.41	6.1 ± 4.22	1.94 ± 2.22
Folate *	Adequate	40.72 ± 7.14	5.65 ± 3.92	1.72 ± 1.96
	Inadequate	48.72 ± 8.54	7.65 ± 5.3	2.72 ± 3.11
Na	Adequate	41.44 ± 7.27	5.76 ± 3.99	1.77 ± 2.03
	Inadequate	42.44 ± 7.44	6.16 ± 4.27	1.97 ± 2.26
K	Adequate	41.3 ± 7.24	5.71 ± 3.95	1.74 ± 1.99
	Inadequate	42.3 ± 7.42	6.11 ± 4.23	1.94 ± 2.22
Ca	Adequate	41.66 ± 7.3	5.85 ± 4.05	1.82 ± 2.08
	Inadequate	42.66 ± 7.48	6.25 ± 4.33	2.02 ± 2.31
P	Adequate	40.95 ± 7.18	5.57 ± 3.85	1.67 ± 1.91
	Inadequate	41.95 ± 7.35	5.97 ± 4.13	1.87 ± 2.14
Mg *	Adequate	38.64 ± 6.77	5.13 ± 3.56	1.46 ± 1.67
	Inadequate	46.64 ± 8.18	7.13 ± 4.94	2.46 ± 2.81
Zn *	Adequate	36.72 ± 6.44	4.65 ± 3.22	1.22 ± 1.39
	Inadequate	44.72 ± 7.84	6.65 ± 4.61	2.22 ± 2.54
Cu	Adequate	41.22 ± 7.23	5.67 ± 3.93	1.73 ± 1.98
	Inadequate	42.22 ± 7.4	6.07 ± 4.21	1.93 ± 2.2
Mn	Adequate	41.85 ± 7.34	5.92 ± 4.1	1.85 ± 2.12
	Inadequate	42.85 ± 7.51	6.32 ± 4.38	2.05 ± 2.35
Fe	Adequate	41.07 ± 7.2	5.61 ± 3.89	1.7 ± 1.94
	Inadequate	42.07 ± 7.38	6.01 ± 4.17	1.9 ± 2.17
I	Adequate	41.31 ± 7.24	5.71 ± 3.95	1.74 ± 2.0
	Inadequate	42.31 ± 7.42	6.11 ± 4.23	1.94 ± 2.22

Values are presented as the mean ± standard deviation (SD) for the BSA (%) at baseline (day 0), day 42, and day 84. Groups were classified according to adequate or inadequate intake of each dietary component prior to treatment initiation. An asterisk indicates a statistically significant difference between groups at the given time point (*p* < 0.05; Student’s *t*-test). LA, linoleic acid; ALA, alpha-linoleic acid; EPA, eicosapentaenoic acid; DHA, docosahexaenoic acid; Na, sodium; K, potassium; Ca, calcium; P, phosphorus; Mg, magnesium; Zn, zinc; Cu, copper; Mn, manganese; Fe, iron; I, iodine.

**Table 8:** Adjusted differences in PASI and BSA between patients with nutrient inadequacy vs. adequacy across treatment time points.

Outcome	Nutrients	Time	Beta (Deficit–Adequate)	95% CI	*p*	q (FDR)
BSA	EPA + DHA	Day 0	10.37	[5.26, 15.47]	**0.0001**	0.0006
		Day 42	3.69	[0.30, 7.09]	**0.0331**	0.0695
		Day 84	3.26	[0.01, 6.51]	**0.0491**	0.0937
	Fiber	Day 0	10.53	[5.77, 15.29]	**0** **.0**	0.0003
		Day 42	5.58	[1.86, 9.31]	**0.0033**	0.0138
		Day 84	4.12	[0.60, 7.64]	**0.0217**	0.0506
	Folate	Day 0	4.61	[−1.04, 10.26]	0.1099	0.1775
		Day 42	−0.14	[−3.58, 3.29]	0.9351	0.9351
		Day 84	−0.52	[−3.15, 2.12]	0.701	0.7748
	Mg	Day 0	4.86	[−1.42, 11.13]	0.1292	0.1938
		Day 42	2.32	[−2.09, 6.74]	0.3025	0.3971
		Day 84	0.47	[−2.63, 3.58]	0.7643	0.8025
	Vitamin A	Day 0	9.16	[4.58, 13.75]	**0.0001**	0.0006
		Day 42	4.14	[0.98, 7.30]	**0.0102**	0.0305
		Day 84	1.66	[−1.03, 4.35]	0.2253	0.3155
	Vitamin D	Day 0	11.97	[4.12, 19.83]	**0.0028**	0.0138
		Day 42	4.49	[0.92, 8.05]	**0.0136**	0.0357
		Day 84	0.66	[−2.03, 3.35]	0.6302	0.7352
	Zn	Day 0	4.9	[−0.94, 10.74]	0.1	0.1751
		Day 42	4.52	[1.18, 7.87]	**0.008**	0.0281
		Day 84	1.56	[−1.83, 4.94]	0.367	0.4534
PASI	EPA + DHA	Day 0	4.1	[1.27, 6.94]	**0.0046**	0.0137
		Day 42	0.37	[−1.18, 1.91]	0.6399	0.7072
		Day 84	1.42	[−0.04, 2.89]	0.0565	0.1094
	Fiber	Day 0	6.04	[3.78, 8.29]	**0** **.0**	0.0
		Day 42	2.51	[0.98, 4.04]	**0.0013**	0.0055
		Day 84	1.66	[−0.05, 3.37]	0.0573	0.1094
	Folate	Day 0	1.86	[−1.08, 4.79]	0.2151	0.3011
		Day 42	0.01	[−1.38, 1.40]	0.9882	0.9882
		Day 84	−0.01	[−1.37, 1.35]	0.988	0.9882
	Mg	Day 0	2.5	[−0.78, 5.78]	0.1348	0.2177
		Day 42	0.74	[−0.90, 2.38]	0.3777	0.4666
		Day 84	0.46	[−1.31, 2.23]	0.6091	0.7072
	Vitamin A	Day 0	5.57	[3.33, 7.81]	**0** **.0**	0.0
		Day 42	2.24	[1.06, 3.42]	**0.0002**	0.0011
		Day 84	2.71	[1.40, 4.01]	**0** **.0**	0.0003
	Vitamin D	Day 0	4.97	[1.15, 8.79]	**0.0108**	0.0283
		Day 42	2.13	[0.75, 3.51]	**0.0024**	0.0086
		Day 84	0.97	[−0.23, 2.18]	0.1141	0.1996
	Zn	Day 0	2.91	[0.08, 5.74]	**0.044**	0.1027
		Day 42	1.0	[−0.43, 2.42]	0.1696	0.2545
		Day 84	0.82	[−0.58, 2.22]	0.2532	0.3323

EPA, eicosapentaenoic acid; DHA, docosahexaenoic acid; Mg, magnesium; Zn, zinc. Values are β coefficients (deficit–adequate) with 95% confidence intervals (CIs), estimated using generalized estimating equations (Gaussian family, exchangeable correlation, cluster-robust SEs). Models included fixed effects for time, nutrient adequacy, and their interaction, adjusted for BMI, age, sex, baseline outcome (PASI0/BSA0), and baseline energy intake. *p* values are two-sided; q values represent false discovery rate (FDR)-adjusted *p*-values (Benjamini–Hochberg) within each outcome family. Statistically significant associations after FDR correction are indicated in bold.

## Data Availability

The original contributions presented in this study are included in the article/supplementary material. Further inquiries can be directed to the corresponding authors.

## Supplementary Materials

The following supporting information can be downloaded at https://www.mdpi.com/article/10.3390/nu17193098/s1: Table S1: Classification of study participants by frequency of consumption of selected food groups; Table S2: Percentage of patients with inadequate intake of macronutrients and selected fatty acids, stratified by sex, with 95% confidence intervals; Table S3: Percentage of patients with inadequate vitamin intake, stratified by sex, with 95% confidence intervals; Table S4: Percentage of patients with inadequate mineral intake, stratified by sex, with 95% confidence intervals; Table S5: Covariate effects from adjusted models of nutrient adequacy and psoriasis severity; Figure S1: Scatter plot of individual PASI values at baseline (day 0), day 42, and day 84 during cyclosporine A treatment; Figure S2: Scatter plot of individual BSA values at baseline (day 0), day 42, and day 84.

## Abbreviations

The following abbreviations are used in this manuscript:

ALA	Alpha-Linolenic Acid
ANOVA	Analysis of Variance
AAS	Atomic Absorption Spectrometry
AI	Adequate Intake
BSA	Body Surface Area
BMI	Body Mass Index
Ca	Calcium
CsA	Cyclosporine A
Cu	Copper
DHA	Docosahexaenoic Acid
EAR	Estimated Average Requirement
EPA	Eicosapentaenoic Acid
Fe	Iron
FFQ	Food Frequency Questionnaire
H_2_O_2_ eq.	Hydrogen Peroxide Equivalent
I	Iodine
K	Potassium
LA	Linoleic Acid
Mg	Magnesium
Mn	Manganese
Na	Sodium
OSI	Oxidative Stress Index
P	Phosphorus
PASI	Psoriasis Area and Severity Index
PUFA	Polyunsaturated Fatty Acid
ROS	Reactive Oxygen Species
SFA	Saturated Fatty Acid
SD	Standard Deviation
TAS	Total Antioxidant Status
TOS	Total Oxidant Status
Zn	Zinc
