# Supplementary material for: Pre-Treatment Nutritional Status as a Predictor of Clinical Outcomes in Moderate-to-Severe Plaque Psoriasis Patients Undergoing Cyclosporine A Therapy
Source: Nutrients. 2025 Sep 29;17(19):3098. doi: 10.3390/nu17193098 (PMC12525798; doi:10.3390/nu17193098)
Supplement: Supplementary file 1 [file nutrients-17-03098-s001.zip › nutrients-3881587-supplementary.pdf]

Supplementary Table S1. Classification of Study Participants by Frequency of Consumption of Selected Food Groups

Food Group No.	Food Group Name	Example Products	Group I (Infrequent Consumption)	Group II (Frequent Consumption)
1	White bread	white wheat bread	≤1 time/week	≥2–3 times/week
2	Wholemeal bread	rye bread	≤1 time/week	≥2–3 times/week
3	Sweets	sweet rolls, cakes, chocolate	≤1 time/week	≥2–3 times/week
4	Flour-based dishes	dumplings, pasta, pancakes	≤1 time/week	≥2–3 times/week
5	Groats/rice/cereals	barley groats, rice, oatmeal	≤1 time/week	≥2–3 times/week
6	Milk and dairy drinks	milk, yoghurt, kefir	≤1 time/week	≥2–3 times/week
7	Cottage cheese	quark	≤1 time/week	≥2–3 times/week
8	Ripened/processed cheese	cheddar, processed cheese	≤1 time/week	≥2–3 times/week
9	Meat	beef, pork	≤1 time/week	≥2–3 times/week
10	Poultry	chicken, turkey	≤1 time/week	≥2–3 times/week
11	Offal	liver, kidneys	≤1 time/week	≥2–3 times/week
12	Sausages	regular sausages	≤1 time/week	≥2–3 times/week
13	High-quality cold cuts	ham, smoked meats	≤1 time/week	≥2–3 times/week
14	Meat products	minced meat products	≤1 time/week	≥2–3 times/week
15	Bacon/lard	fatback, lard	≤1 time/week	≥2–3 times/week
16	Canned meat	all types	Excluded	Excluded
17	Canned fish	tuna, sardines	≥1 time/week	≥2–3 times/week
18	Fresh fish	salmon, cod	≥1 time/week	≥2–3 times/week
19	Eggs	hen eggs	≤1 time/week	≥2–3 times/week
20	Butter	butter	≤1 time/week	≥2–3 times/week
21	Margarine	soft margarine	≤1 time/week	≥2–3 times/week
22	Vegetable oils	sunflower oil, rapeseed oil	≤1 time/week	≥2–3 times/week

23	Olive oil	olive oil	≤1 time/week	≥2–3 times/week
24	Potatoes	boiled potatoes	≤1 time/week	≥2–3 times/week
25	Raw vegetables	cucumber, tomato	≤1 time/week	≥2–3 times/week
26	Cooked vegetables	boiled carrots, cabbage	≤1 time/week	≥2–3 times/week
27	Fruit	apples, berries	≤1 time/week	≥2–3 times/week
28	Fruit juices/drinks	orange juice, apple juice	≤1 time/week	≥2–3 times/week
29	Jam	all types	≤1 time/week	≥2–3 times/week
30	Honey	honey	≤1 time/week	≥2–3 times/week
31	Sugar in beverages	sugar in tea or coffee	≤1 time/week	≥2–3 times/week
32	Legume products	beans, lentils	≤1 time/week	≥2–3 times/week
33	Beer	beer	≤1 time/week	≥2–3 times/week
34	Wine	wine	Excluded	Excluded
35	Vodka	vodka	Excluded	Excluded
36	Tea	black tea	≤1 time/week	≥2–3 times/week
37	Coffee	coffee	≤1 time/week	≥2–3 times/week

Supplementary Table S2. Percentage of patients with inadequate intake of macronutrients and selected fatty acids, stratified by sex, with 95% confidence intervals

Nutrient	Women (%) [95% CI]	Men (%) [95% CI]
Protein	41 (21.6–64.0)	32 (14.5–51.9)
Fat	75 (52.7–90.4)	89 (69.9–97.2)
Digestible carbohydrates	21 (9.6–47.3)	29 (14.5–51.9)
Fiber	69 (46.9–86.7)	93 (76.4–99.1)
LA	76 (52.7–90.4)	88 (69.9–97.2)
ALA	67 (41.3–82.7)	95 (76.4–99.1)
EPA+DHA	91 (65.7–96.7)	80 (58.4–91.9)

LA, linoleic acid; ALA, alpha-linolenic acid; EPA, eicosapentaenoic acid; DHA, docosahexaenoic acid. Values represent the percentage of patients with intake below the dietary reference values (EAR or AI). Confidence intervals (95% CI) were calculated using the Wilson method.

Supplementary Table S3. Percentage of patients with inadequate vitamin intake, stratified by sex, with 95% confidence intervals

Vitamin	Women (%) [95% CI]	Men (%) [95% CI]
A	52 (31–72)	29 (14–51)
E	41 (22–64)	14 (5–34)
D	5 (1–23)	7 (2–24)
B1	38 (20–61)	32 (16–54)
B2	15 (6–36)	17 (7–37)
B6	21 (9–43)	19 (8–39)
B12	34 (17–56)	33 (16–55)
PP	10 (3–29)	2 (0–17)
C	40 (21–64)	31 (15–53)
Folates	81 (58–93)	89 (70–97)

Values represent the percentage of patients with intake below the dietary reference values (EAR or AI). Confidence intervals (95% CI) were calculated using the Wilson method.

Supplementary Table S4. Percentage of patients with inadequate mineral intake, stratified by sex, with 95% confidence intervals

Mineral	Women (%) [95% CI]	Men (%) [95% CI]
Na	53 (32–72)	51 (31–71)
K	39 (20–62)	37 (19–59)
Ca	88 (66–97)	60 (39–79)
P	2 (0–17)	4 (1–20)
Mg	54 (33–74)	64 (42–82)
Zn	41 (22–64)	29 (14–51)
Cu	32 (15–55)	27 (13–48)
Mn	91 (69–98)	94 (74–99)
Fe	21 (9–43)	9 (3–26)
I	43 (23–66)	34 (17–57)

Na, sodium; K, potassium; ca, calcium; P, phosphorus; Mg, magnesium; Zn, zinc; Cu, copper; Mn, manganese; Ge, iron; I, iodine

Values represent the percentage of patients with intake below the dietary reference values (EAR or AI). Confidence intervals (95% CI) were calculated using the Wilson method.

Supplementary Table S5. Covariate effects from adjusted models of nutrient adequacy and psoriasis severity.

Outcome	Nutrients	Term	Beta	95% CI	p
PASI	Fiber	BMI	0.307	[0.084, 0.531]	0.007
		age	0.081	[-0.012, 0.174]	0.088
		male	1.404	[-0.497, 3.305]	0.148
		energy	0.001	[-0.001, 0.002]	0.476
		PASI	0.948	[0.738, 1.158]	<0.001
	EPA+DHA	BMI	0.052	[-0.201, 0.304]	0.689
		age	0.034	[-0.072, 0.140]	0.533
		male	1.999	[-0.189, 4.188]	0.073
		energy	0.001	[-0.001, 0.003]	0.400
		PASI	0.949	[0.691, 1.208]	<0.001
	Vitamin A	BMI	0.037	[-0.188, 0.263]	0.745
		age	0.054	[-0.004, 0.112]	0.070
		male	2.121	[0.576, 3.666]	0.007
		energy	0.001	[-0.001, 0.003]	0.232
		PASI	1.041	[0.841, 1.240]	<0.001
	Vitamin D	BMI	0.105	[-0.135, 0.345]	0.390
		age	0.023	[-0.079, 0.125]	0.655
		male	2.271	[0.051, 4.491]	0.045
		energy	0.001	[-0.001, 0.003]	0.308
		PASI	0.934	[0.665, 1.203]	<0.001
	Folate	BMI	0.086	[-0.164, 0.335]	0.502
		age	0.034	[-0.082, 0.150]	0.564
		male	2.330	[0.109, 4.552]	0.040

		energy	0.001	[-0.001, 0.003]	0.518
		PASI	0.990	[0.738, 1.242]	<0.001
	Mg	BMI	0.064	[-0.188, 0.316]	0.619
		age	0.030	[-0.073, 0.133]	0.571
		male	1.977	[-0.293, 4.247]	0.088
		energy	<0.001	[-0.002, 0.003]	0.711
		PASI	0.969	[0.691, 1.247]	<0.001
	Zn	BMI	0.080	[-0.168, 0.329]	0.526
		age	0.027	[-0.081, 0.136]	0.620
		male	2.241	[0.061, 4.420]	0.044
		energy	<0.001	[-0.002, 0.002]	0.873
		PASI	0.988	[0.730, 1.246]	<0.001
BSA	Fiber	BMI	0.744	[0.348, 1.141]	<0.001
		age	0.170	[-0.010, 0.350]	0.064
		male	2.621	[-1.734, 6.977]	0.238
		energy	<0.001	[-0.004, 0.003]	0.805
		BSA	0.832	[0.557, 1.108]	<0.001
	EPA+DHA	BMI	0.192	[-0.209, 0.592]	0.348
		age	0.072	[-0.125, 0.268]	0.475
		male	3.541	[-0.407, 7.489]	0.079
		energy	<0.001	[-0.003, 0.004]	0.927
		BSA	0.853	[0.564, 1.141]	<0.001
	Vitamin A	BMI	0.279	[-0.134, 0.692]	0.185
		age	0.101	[-0.043, 0.244]	0.168

	male	3.442	[-0.632, 7.515]	0.098
	energy	0.001	[-0.003, 0.004]	0.671
	BSA	0.890	[0.612, 1.167]	<0.001
Vitamin D	BMI	0.337	[-0.047, 0.721]	0.085
	age	0.056	[-0.120, 0.232]	0.533
	male	4.512	[-0.140, 9.163]	0.057
	energy	<0.001	[-0.004, 0.004]	0.886
	BSA	0.816	[0.505, 1.127]	<0.001
Folate	BMI	0.355	[-0.065, 0.775]	0.097
	age	0.089	[-0.125, 0.303]	0.416
	male	4.489	[-0.152, 9.129]	0.058
	energy	-0.001	[-0.004, 0.003]	0.780
	BSA	0.765	[0.461, 1.069]	<0.001
Mg	BMI	0.285	[-0.129, 0.698]	0.178
	age	0.074	[-0.112, 0.260]	0.434
	male	3.752	[-0.899, 8.402]	0.114
	energy	-0.001	[-0.005, 0.003]	0.651
	BSA	0.798	[0.504, 1.092]	<0.001
Zn	BMI	0.363	[-0.057, 0.782]	0.090
	age	0.085	[-0.110, 0.279]	0.392
	male	4.639	[0.293, 8.984]	0.036
	energy	-0.003	[-0.007, 0.001]	0.175
	BSA	0.683	[0.403, 0.963]	<0.001

Values are β coefficients (95% CI, *p*) estimated from generalized estimating equations (Gaussian family, exchangeable correlation, cluster-robust SEs by subject). Each nutrient model was adjusted for the following covariates: body mass index (BMI, kg/m^2), age (years), sex (male vs. female), baseline

disease severity (PASI0 for PASI models; BSA0 for BSA models), and baseline energy intake (kcal/day).
EPA, Eicosapentaenoic acid; DHA, Docosahexaenoic acid; Mg, magnesium; Zn, zinc

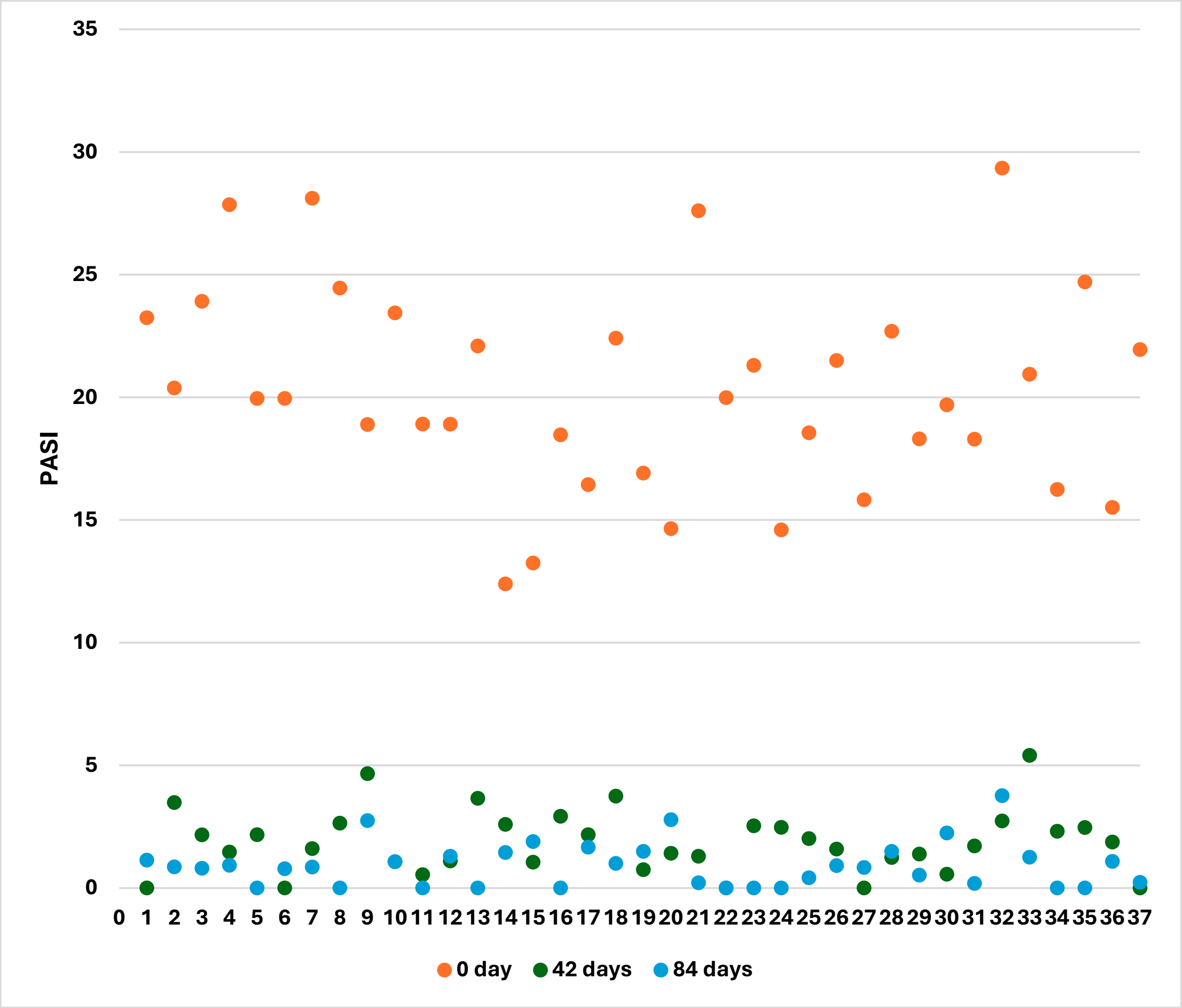

Supplementary Figure S1. Scatter plot of individual PASI values at baseline (day 0), day 42, and day 84 during cyclosporine A treatment (n = 37).

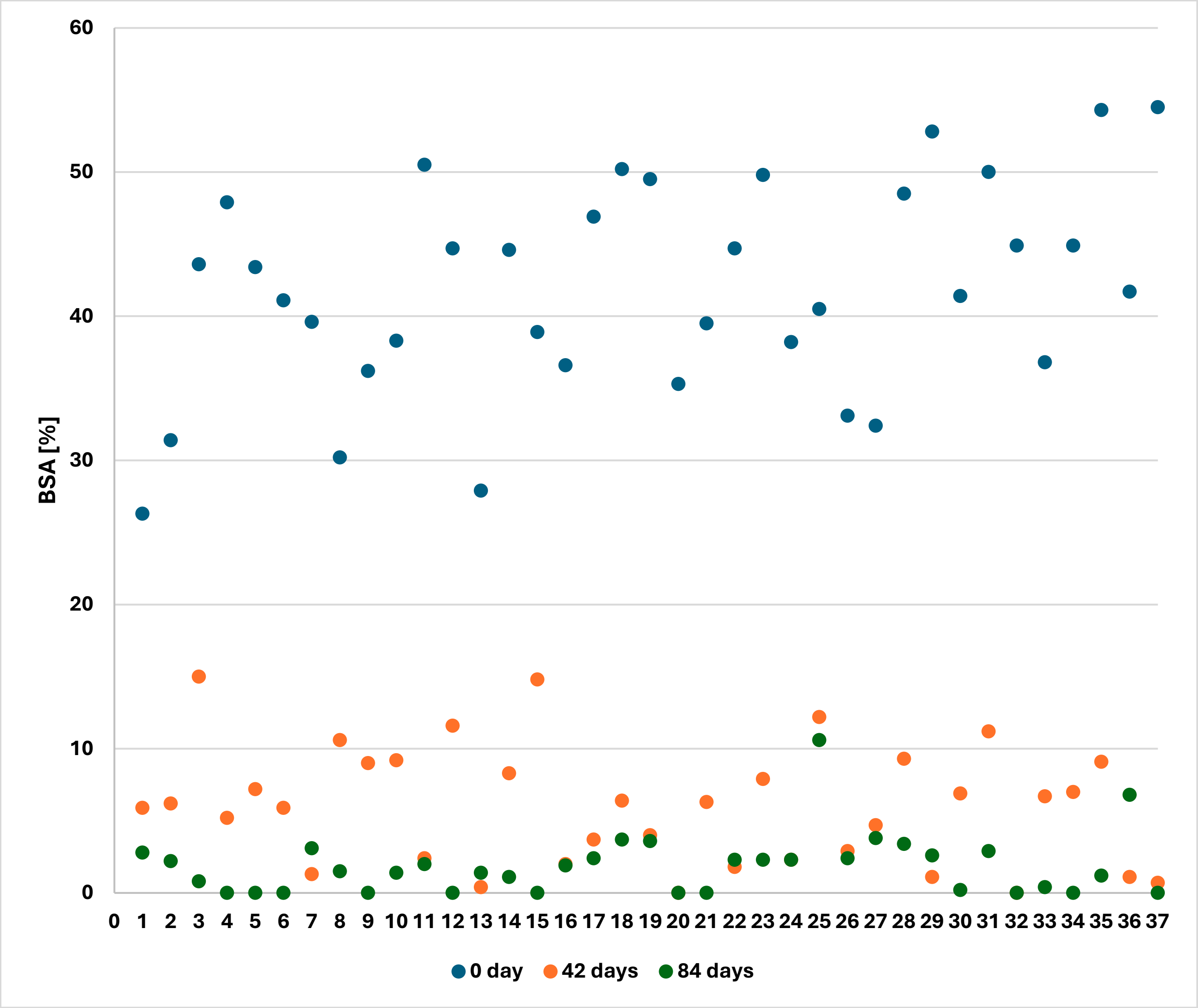

Supplementary Figure S2. Scatter plot of individual BSA values at baseline (day 0), day 42, and day 84.
